# Strengthening primary HIV prevention: better use of data to improve programmes, develop strategies and evaluate progress

**DOI:** 10.1002/jia2.25538

**Published:** 2020-06-30

**Authors:** James R Hargreaves, Judith D Auerbach, Bernadette Hensen, Saul Johnson, Simon Gregson

**Affiliations:** ^1^ Faculty of Public Health and Policy London School of Hygiene and Tropical Medicine London United Kingdom; ^2^ Department of Medicine University of California San Francisco San Francisco CA USA; ^3^ Faculty of Infectious and Tropical Diseases London School of Hygiene and Tropical Medicine London United Kingdom; ^4^ Health Practice Genesis Analytics Johannesburg South Africa; ^5^ Faculty of Medicine Imperial College London London United Kingdom

**Keywords:** HIV prevention, primary prevention, cascades, HIV prevention programmes, data, programme monitoring

It is heartening to learn from recent data in multiple settings that decreasing community viral load through “universal test and treat” (UTT) programmes is having a significant impact on HIV morbidity, mortality and the rate of new HIV infections in some settings [1, 2, 3. However, focusing only on preventing *transmission* from a person already living with HIV to one who is not, is only half of the equation and will by itself not “end HIV.” A crucial focus remains preventing *acquisition* of HIV infection among people at risk. Achieving this requires that we strengthen primary HIV prevention programmes because HIV incidence declines attributable to treatment may be slower than required to meet global goals [4]; treatment as prevention may have less impact where a high proportion of transmission involves those in early stage HIV infection [5; and, critically, because it is essential that individuals and communities have the autonomy to avoid acquiring HIV if at all possible.

Happily, the range of efficacious tools for primary prevention of HIV infection has increased in recent years. These include condoms, voluntary medical male circumcision, oral pre‐exposure prophylaxis (PrEP), clean needles and associated drug use paraphernalia, as well as a range of behaviours, such as information sharing between partners about HIV serostatus, use of antiretroviral therapy (ART), or HIV viral load before making decisions about sex and drug‐using activities. However, optimism must be tempered by the fact that, although some tools have been with us for some time, their population impact has been limited by individual, interpersonal, social (including cultural, economic and political) and structural factors.

The aim of a primary HIV prevention programme is to increase the uptake and continued use of efficacious HIV prevention tools and other safe behaviours among those who may be at risk of infection. Achieving this requires strategies that are appropriate, acceptable and reach those in need. Programmers can only know how well they are doing in this regard by collecting and using data in a systematic way. This special issue of *Journal of the International AIDS Society* showcases current thinking on how data can be used to support decision makers in deploying their resources to maximize the impact of primary HIV prevention programmes.

Formulating an HIV prevention strategy includes a range of considerations: whether and how to focus efforts to particular populations versus implementing a general population approach; which prevention tools to offer; and, the extent to which the programme should focus on strengthening motivation for HIV prevention behaviours, improving supply channels, and/or supporting the capacity of individuals to enact HIV avoidance behaviours. Data‐informed insights are needed to support these decisions. The data must go beyond an assessment of where and among whom new HIV infections are occurring. Only by understanding the modifiable determinants of risk and barriers to prevention can programmers select, implement, monitor and strengthen the most appropriate interventions and policies.

This is not a new call. More than 10 years ago, "know your epidemic, know your response" was positioned as a "rallying cry" for an intensified HIV prevention response [6. The need to renew this message periodically reflects the reality that the measurement challenges we face in HIV prevention are formidable, the determinants of HIV risk are multiple, complex and interacting, and the barriers to sustained behaviour change significant. It is clear that interdisciplinary research and data‐driven multi‐sectoral planning remain critical to strengthening primary HIV prevention. The papers in this special issue reflect the effort, innovation, and challenges faced by those who share this vision today.

In responding to our call for papers on "Data‐driven HIV prevention," many (though not all) of the papers attempt to operationalize an HIV prevention cascade. HIV prevention cascades are a promising framework that can be used to generate insights from data in many instances. We were pleased to see the innovation and thought reflected in the papers that provided cascade models: nevertheless, there remains work to do. As Auerbach *et al*. [7 outline, debates about the merits and pitfalls of HIV prevention cascades are ongoing, but there is general agreement that a standardized programme monitoring tool (like the treatment cascade) would be helpful. As the authors note, emerging consensus identifies the core steps of primary prevention cascade models for programme monitoring and research as first characterizing the priority population at risk, and then tracking motivation, access, uptake and/or effective use of prevention tools among this population. We use this prevention cascade structure to provide a brief overview of the content of this special issue.

Identifying priority populations for whom primary prevention efforts are to be strengthened is the first job for any HIV prevention strategy. Rice *et al*. [8 reflect on a pilot of tests of recent HIV infection in diverse routine HIV testing settings in Kenya and Zimbabwe and consider the potential use of these tests to help focus prevention activities. Virkud *et al*. [9 generate cascades that show the need for HIV prevention to be strengthened among those who visit bars, hotels and guest houses in cross‐border areas in East Africa. Sibanda *et al*. [10 show that new HIV infections among pregnant mothers are a critical driver of infant infections in Zimbabwe and highlight the need to strengthen prevention cascades among HIV‐negative women.

HIV prevention programmes seek to increase individuals’ motivation to undertake behaviours that will protect them and others from HIV infection. [11 The HIV prevention cascade recognizes that the range of relevant behaviours include decisions such as to avoid sex, take PrEP, suggest condom use to a sexual partner, and be circumcised. An individual’s behavioural intention is also influenced by perceived social norms. Hill *et al*. [12 present data from one priority population – adolescent girls and young women (AGYW) in Malawi – and carefully examine the relationships between risk perception, “epidemiological” risk, and the motivation of these young women to take PrEP. They conclude that motivation remains lower than optimal and more efforts are needed as PrEP rolls out. Similarly, Ramautarsing *et al*. [13 used programmatic data to document PrEP roll‐out among transgender women and men who have sex with men (MSM) in Thailand. They found that the biggest gap in the cascade for both population groups was in demand: many clients who were offered PrEP did not initiate PrEP because they did not perceive themselves to be at risk for HIV acquisition.

When people are motivated to use existing HIV prevention methods, lack of access to them can have population‐level impacts on infection rates. The implications of poor access are shown by a modelling exercise of couples’ voluntary counselling and testing programmes, which can facilitate prevention choices, in six African countries presented by Wall *et al*. [14 When new methods, such as PrEP, are introduced, gains in HIV prevention can be made through strengthening supply channels and breaking down access barriers; but uptake takes time and is influenced by attitudes and behaviours of providers and clients. Were *et al*. [15 use data from the first two years of PrEP roll‐out in Kenya to construct prevention cascades and to highlight missed opportunities in PreP delivery and uptake among three priority populations – female sex workers (FSW), MSM and AGYW. For AGYW, the biggest missed opportunity was screening. For MSM and FSW, the biggest missed opportunity was that, among those who were screened and found eligible for PrEP, the majority did not initiate PrEP despite its availability.

Even when people are motivated, have access to, and initiate HIV prevention measures, social and structural barriers may impede their capacity to consistently use them. Programmatic innovation in addressing these barriers remains critical. Chabata *et al*. [16 show that knowledge of condom efficacy is high and availability good among young women who sell sex in Zimbabwe, and yet consistent use is low, especially among those young women who recently experienced violence from a sexual partner. Holmes *et al*. [17 characterize the relationship environments of young women in South Africa and how these influence PrEP use/adherence, secondary distribution of HIV self‐tests to partners, and of sharing information about HIV status. In a modelling study, Bershteyn *et al*. [18 demonstrate how implementation challenges along the prevention cascade differentially influence the population‐level impacts of the use of oral PrEP and long‐acting PrEP in Kenya. Wilson *et al*. [19 report on social and structural determinants and patterns of PrEP use among two sexual minority populations – transgender women and MSM in the United States. They find differences in the PrEP cascades for the two populations, with transgender women being more affected by social‐structural issues of poverty, homelessness and unemployment than MSM. Their paper underscores the need to distinguish and specify priority populations, and to identify the particular HIV prevention gaps, barriers and approaches relevant to each.

As Auerbach *et al*. [7 note, and the aforementioned examples attest, while the cascade model has proven to be useful for monitoring progress and gaps in HIV prevention programming in many settings, it does have limitations. Dumchev *et al*. [20 present an analysis of data from an integrated bio‐behavioural survey in Ukraine to assess the HIV prevention cascade for people who inject drugs (PWID). They find that in their context there was little consistency between their “access to services” and “effective use” measures, given that people who inject drugs often obtain sterile syringes from sources other than the programmes being monitored.

Across the papers included in this special issue, authors are striving for a strengthened feedback loop, from data to programming decisions, for primary HIV prevention to support implementers and managers to deploy the interventions that are most needed to address the determinants of risk in their settings. Most papers use existing data streams to populate their cascade models, and many identify significant measurement and interpretation challenges in operationalizing key elements of the cascade. Further innovation remains essential to strengthen our capacity to track cascades and thereby strengthen the right intervention mix. Generally, it is not feasible to create new data sources or make fundamental changes to existing data sources to inform prevention programming. However, more work would be useful to establish the extent to which minor changes to routine data systems, including further integration of qualitative enquiry, would be feasible for different settings, populations and methods that would improve the validity and utility of the cascades that can be generated.

Most of the papers submitted for the special issue focussed on single methods of prevention. To some extent this may reflect the continuing siloing of programmes for different prevention methods despite the common call for combination prevention approaches [21. In principle, it is quite possible to create HIV prevention cascades for combination prevention [5 and we would encourage more attempts to do this. We would also like to see a greater effort to bring HIV prevention cascade thinking into modelling efforts that often guide programme decision making. Again, a greater focus on qualitative and participatory data enquiry that unpacks the reasons for drop offs in the cascade, could also accelerate the loop from data to programmatic improvement.

We applaud the authors of the papers in this series for grappling with some thorny issues in primary HIV prevention data collection and, particularly, cascade analysis. We hope readers find this special issue helpful in their own efforts to strengthen ongoing monitoring, evaluation and advocacy of HIV prevention to meet global goals by 2030.

## COMPETING INTERESTS

The authors have no competing interests to declare.

## AUTHORS’ CONTRIBUTIONS

JRH drafted the initial manuscript. JDA provided substantial revisions and finalized the draft manuscript. All authors critically reviewed the manuscript, suggested revisions and editorial changes, and approved the final version.
